# SIRT1 Attenuates Kidney Disorders in Male Offspring Due to Maternal High-Fat Diet

**DOI:** 10.3390/nu11010146

**Published:** 2019-01-11

**Authors:** Long T. Nguyen, Crystal H. Mak, Hui Chen, Amgad A. Zaky, Muh G. Wong, Carol A. Pollock, Sonia Saad

**Affiliations:** 1 Renal medicine, Kolling Institute, Royal North Shore Hospital, University of Sydney, Sydney, New South Wales 2065, Australia; hui.chen-1@uts.edu.au (H.C.); amgadadolf@hotmail.com (A.A.Z.); muhgeot.wong@sydney.edu.au (M.G.W.); carol.pollock@sydney.edu.au (C.A.P.); sonia.saad@sydney.edu.au (S.S.); 2 School of Life Sciences, Faculty of Science, University of Technology Sydney, Sydney, New South Wales 2007, Australia; Crystal.H.Mak@student.uts.edu.au

**Keywords:** Obesity, chronic kidney disease, foetal programming, sirtuin

## Abstract

Maternal obesity has been associated with kidney disorders in male offspring. Our previous studies have demonstrated that Sirtuin (SIRT)1, an essential regulator of metabolic stress responses, is suppressed in the offspring as the result of maternal high-fat diet (HFD) consumption, which is likely to underpin the adverse metabolic and renal outcomes. To examine if SIRT1 overexpression or activation early in life can protect the offspring kidney, wild-type (WT) and transgenic (Tg) offspring were born to the same diet-induced obese female C57BL/6 mice through breeding with hemizygous SIRT1-transgenic (Tg) male mice and examined for renal pathological changes. In separate experiments, SIRT1 activator SRT1720 (25 mg/kg/2 days i.p) was administrated in WT offspring over 6 weeks of postnatal high-fat diet exposure. The results show that offspring born to obese dams have increased kidney weight, higher levels of renal triglycerides, and increased expression of oxidative stress, inflammatory, and fibrotic markers, as well as increased albuminuria compared to offspring of control dams. Both SIRT1 overexpression and SRT1720 treatment attenuated renal lipid contents and expression of lipogenesis, oxidative stress, and inflammatory markers; however, fibrosis was modestly reduced and albuminuria was not affected. The findings suggest that SIRT1 therapy can ameliorate some pathological mechanisms of kidney programming due to maternal obesity but may not be sufficient to prevent the resulting chronic kidney injury.

## 1. Introduction

Obesity is a global health concern due to its prevalence and impact on the development of various diseases such as type 2 diabetes and hypertension, as well as secondary comorbidities including chronic kidney diseases (CKD). In obese people, the levels of circulating lipid and fatty acids are much higher than in non-obese individuals and can thus accumulate into non-adipose tissues such as heart, liver, pancreas, and kidney. Renal lipid accumulation or ‘lipotoxicity’ has been suggested to be the direct cause of structural and functional changes in mesangial cells, podocytes, and tubular cells [1,2], leading to obesity-related nephropathy [3]. Additionally, obesity-mediated diabetes and hypertension also contribute to the development of CKD. 

Recent evidence suggests that the effects of obesity on CKD can also be intergenerational due to intrauterine foetal programming. Maternal obesity and high-fat diet consumption during pregnancy have been found to not only result in metabolic disorders such as glucose intolerance, insulin resistance, hyperlipidaemia, and hepatic steatosis [4,5,6,7], but also disrupt renal lipid metabolism and induce oxidative stress [8], albuminuria, glomerulosclerosis, and tubulointerstitial fibrosis in the offspring [9]. The molecular mechanisms and mediating factors of such intergenerational effects are still poorly understood.

Our recent studies suggest that Sirtuin (SIRT)1, an essential regulator of metabolism and stress responses [10,11], may play an important role in maternal obesity-induced foetal programming [12], particularly metabolic disorders such as insulin resistance and hepatic steatosis [8,13]. In kidney, we have shown in a rat model that SIRT1 expression is suppressed in male offspring born to obese dams in association with increased renal lipid accumulation [8]. SIRT1 is known to promote lipid catabolism in muscle and liver by activating peroxisome proliferator-activated receptor gamma coactivator 1 alpha (PGC-1α) [14,15], an essential regulator of mitochondria biosynthesis and fatty acid oxidation, while suppressing de novo lipogenesis through sterol regulatory element-binding protein (SREBP)-1c, carbohydrate-responsive element-binding protein (ChREBP), and peroxisome proliferator-activated receptor gamma (PPARγ) [16,17], leading to attenuated lipotoxicity. SIRT1-deficient mice have elevated kidney inflammation [18] and urinary albumin excretion [19]. SIRT1 activation by SRT1720 increases the expression of antioxidant enzymes including superoxide dismutase (SOD), glutathione peroxidase (GPx-1), and glutathione, leading to attenuated levels of fibrotic injury due to unilateral ureteral obstruction (UUO) [20]. With regard to CKD, SIRT1 activation by resveratrol increases (SOD)2 expression and reduces the levels of oxidative stress markers 8-OHdg and nitrotyrosine in a mouse model of type 2 diabetes [21]. Likewise, SIRT1 transgenic mice demonstrate ameliorated diabetic nephropathy [19]. 

As such, we hypothesised that upregulation of SIRT1 signalling at early postnatal ages will improve the male offspring’s renal outcomes. In this study, using two different models, we confirm the protective roles of SIRT1 in foetal kidney programming due to maternal obesity.

## 2. Methods

### 2.1. Animals

The study was approved by the Animal Care and Ethics Committee of the University of Sydney (RESP/15/22). All methods were performed in accordance with the relevant guidelines and regulations in the Australian Code of Practice for the Care and Use of Animals for Scientific Purposes. Female C57BL/6 mice (8 weeks) or SIRT1 transgenic (SIRT1-Tg) mice were fed a high-fat diet (HFD, 20 kJ/g, 43.5% calorie as fat, Specialty Feed, Glen Forrest, WA, Australia) or standard rodent chow (11 kJ/g, 14% calorie as fat, Gordon’s Speciality Stockfeeds, Yanderra, NSW, Australia) for 6 weeks before mating and throughout gestation and lactation [6]. As our previous showed sex-specific regulation of SIRT1 by maternal HFD, all female mice were culled on postnatal day (P) 1 and male mice were adjusted to 4-6 pups/ litter. 

**Figure 1:**
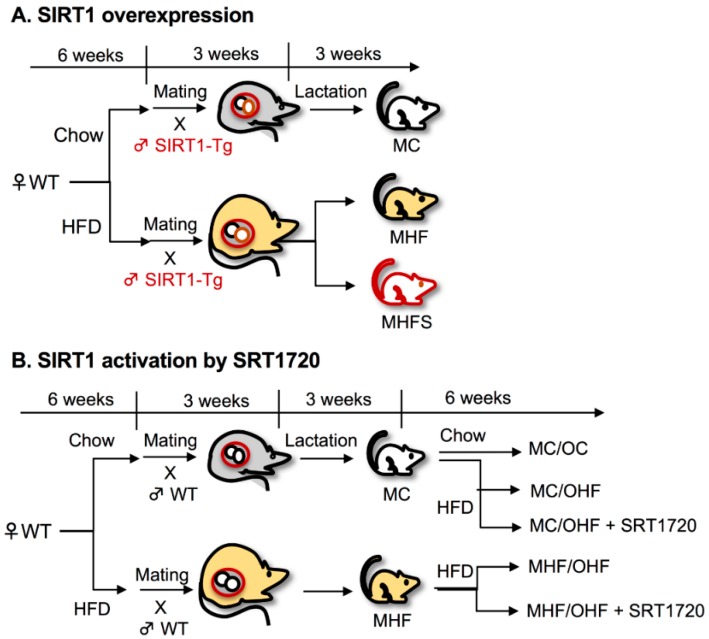
Animal models of SIRT1 overexpression (**A**) and activation (**B**) in offspring. SIRT: sirtuin; MC: offspring of chow-fed dams; MHF: offspring of HFD-fed dams; MHFS: MHF offspring with SIRT1 overexpression. OC: chow-fed offspring; OHF: HFD-fed offspring; WT: wild-type; Tg: transgenic, HFD: high-fat diet.

In the first experiments, wild-type (WT) female mice were mated with Tg male mice to produce both WT and Tg offspring without maternal genotypic modification (Figure 1A). Three main groups were studied: WT offspring born to control dams (MC, *n* = 20), WT offspring born to HFD-fed dams (MHF, *n* = 26), and Tg offspring born to HFD-fed dams (MHFS, *n* = 11). The original Tg colony was a generous gift from Dr. Lindsay Wu (University of New South Wales, Australia). Mice were genotyped in accordance to the Jackson Laboratory genotyping protocol for the B6.Cg-Col1a1^tm1(CAG-Sirt1)Dsin^/Mmjax strain using crude DNA extracted with DirectPCR Lysis Reagent (Mouse Tail) (Viagen Biotech, California, USA) and culled at weaning (P20) for kidney studies. In the second experiments, male offspring from chow or HFD-fed WT breeders (MC and MHF, respectively) were fed chow or HFD from P20 to week 9 (OC and OHF, respectively). Animals born to HFD-fed dams and/or fed on HFD themselves were administrated a low dose of SIRT1 activator SRT1720 (S, 25 mg/kg/2 days i.p, Selleckchem, Houston, TX, USA) or vehicle control (Figure 1B). The experimental design resulted in five groups: MC/OC (*n* = 9), MC/OHF (*n* = 17), MHF/OHF (*n* = 15), MC/OHF/S (*n* = 9), and MHF/OHF/S (*n* = 9). These offspring were culled at week 9. All pups were deeply anaesthetised with 3% isoflurane and euthanised upon cardiac puncture for blood collection after 5h fasting. Phosphate-buffered saline (PBS, 1%) was used for whole body perfusion. Tissues were snapped frozen and stored at −80 °C or fixed in neutral buffered formalin (10%) for approximately 36 h for later analyses.

### 2.2. Urine Collection and Urinary Albumin Creatinine Ratio Analysis

The urine of P20 offspring was collected directly from the bladder during culling, while that of week 9 offspring was collected after a 24-h stay in metabolic cages. The urine was stored in −80 °C and examined for creatinine (Cayman, MI, USA) and albumin (Abcam, VIC, Australia) according to the manufacturers’ instructions. Urinary albumin creatinine ratio (UACR) was used to account for potential differences in hydration in the animal cohort.

### 2.3. Protein and Lipid Extraction from Tissues 

The tissues were homogenized in Triton X-100 lysis buffer (pH 7.4, 150 mM NaOH, 50 mM Tris-HCl, 1% Triton X-100, Roche protease inhibitor) using TissueRuptor (Qiagen, Hilden, Germany). Lipid and protein were extracted and the concentration was measured according to our previously published protocols [8] using Roche triglyceride reagent GPO-PAP (Roche Life Science, NSW, Australia) and Pierce BCA Protein Assay Kit (Thermo Scientific, VIC, Australia) according to the manufacturer’s instructions. Lipid concentrations were normalised to the weight of tissue homogenized. Protein concentrations were standardised to 5 μg/μL.

### 2.4. Quantitative RT-PCR 

Total RNA of renal tissues was isolated using RNeasy Plus Mini Kit (Qiagen Pty Ltd., CA, USA) according to the manufacturer’s instructions, while fat tissues and hypothalamus’s RNA were extracted using Trizol Reagent (Sigma-Aldrich). The purified total RNA was used as a template to generate first-strand cDNA using the First Strand cDNA Synthesis Kit (Roche Life Science, NSW, Australia). The amplicons of target genes were amplified with SYBR Green probes (iTaq Universal SYBR Green Supermix, Bio-Rad, NSW, Australia) using an ABI Prism 7900 HT Sequence Detection System (Applied Biosystems, CA, USA). Primers were as per our previous publications [8,13]. Gene expression was standardized to β-actin mRNA and log-transformed.

### 2.5. Immunoblotting 

The proteins were electrophoresed and electroblotted onto the Hybond nitrocellulose membrane (Amersham Pharmacia Biotech, Amersham, UK), which were then incubated with a primary antibody at 4 °C overnight. The primary antibodies used for immunoblotting included manganese superoxide dismutase (MnSOD, rabbit, dilution 1:2000, EMD Millipore, North Ryde, NSW, Australia) and GPx-1 (goat, dilution 1:500, R&D System, Minneapolis, MN, USA). β-actin (goat, dilution 1:3000, Santa Cruz, TX, USA) was used as the housekeeping protein. Subsequently, the membrane were incubated with a horseradish peroxidase-conjugated secondary antibodies (1:5000, Cell Signalling, MA, USA). The immunoblots were developed by adding the Luminata Western HRP Substrates (Millipore, MA, USA) to the membrane and exposed for an appropriate duration using ImageQuant LAS 4000 (Fujifilm, Tokyo, Japan). ImageJ (National Institutes of Health, USA) was used for densitometric analyses.

### 2.6. SIRT1 Activity Assay

Nuclear protein was extracted according to the protocol ‘Nuclear protein extraction without the use of detergent (Sigma-Aldrich, Dublin, Ireland) using a hypotonic lysis buffer (10 mM 4-(2-hydroxyethyl)-1-piperazineethanesulfonic acid (HEPES), pH 7.9, with 1.5 mM MgCl2 and 10 mM KCl, 1 mM Dithiothreitol), followed by centrifuge to separate nuclear and cytoplasmic fractions. The nuclear proteins were extracted using extraction buffer (20 mM HEPES, pH 7.9, with 1.5 mM MgCl2, 0.42 M NaCl, 0.2 mM EDTA, 25% (v/v) glycerol, 1 mM Dithiothreitol). No protease inhibitor was used in the extraction to avoid interference with SIRT1 activity measurement. SIRT1 activity was then measured using the SIRT1 activity assay kit (Abcam, Cambridge, UK) as per manufacturer’s instruction.

### 2.7. Immunohistochemistry

Immunohistochemistry (IHC) staining was performed as previously described [8]. Briefly, tissues were fixed in 10% formalin for 36-h and embedded in paraffin or frozen-embedded in OCT solution (Tissue-Tek). Paraffin sections were prepared at a 4-μm thickness and mounted on microscope slides (Trajan Scientific and Medical, VIC, Australia). Antigen retrieval was performed at 99 °C for 20 min in 0.01 M, pH 6.0 citric buffer. Endogenous peroxidase was deactivated with 3% H2O2 (Sigma-Aldrich, Dublin, Ireland). The slides were then blocked by Protein Block Serum-Free (Dako, Glostrup, Denmark), and incubated with one of the primary antibodies, which included Fibronectin, Collagen type I (dilution 1:750, Abcam, Cambridge, UK), and 8-hydroxy-2′ -deoxyguanosine (8-OHdg, dilution 1:200, Cell Signalling Technology, MA, USA). After overnight incubation at 4 °C, biotinylated secondary anti-rabbit IgG antibodies (Dako) were incubated for 30 mins and finally horseradish peroxidase (HRP)-conjugated streptavidin (Dako) for 10 mins. Using a light microscope (Leica DM750 photomicroscope with ICC50W digital camera), six consecutive non-overlapping fields from each kidney section were photographed at 20× magnification. Image J (National Institutes of Health, USA) was used to quantitate the staining area percentage.

### 2.8. Statistical Analysis

Data are expressed as column (mean ± SEM) or box plots (25th to 75th percentiles, whisker extends from the minimum to the maximum value). One-way ANOVA followed by Bonferroni post-hoc tests were used.

## 3. Results

### 3.1. SIRT1 Overexpression Suppresses Renal Lipid Accumulation in MHF Offspring

**Figure 2:**
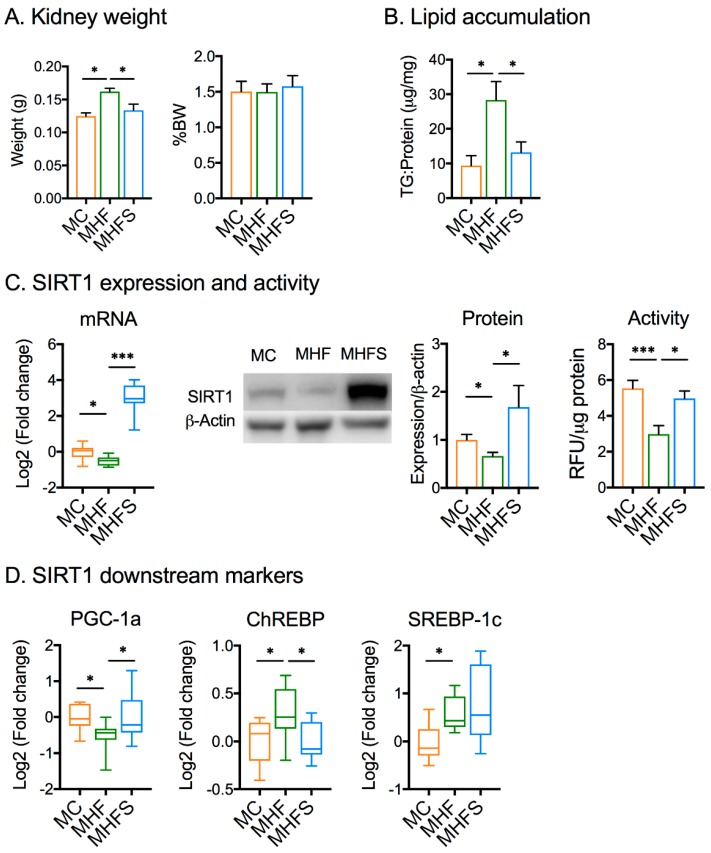
SIRT1 overexpression reduces kidney weight and lipid accumulation in offspring born to obese dams. (**A**) Kidney weight (*n* = 11–26). (**B**) Kidney triglyceride level (*n* = 6). (**C**) SIRT1 expression and activity (*n* = 6). (**D**) The expression of downstream markers of SIRT1 (*n* = 6). Data are expressed by mean ± SEM or box plot (min to max, the central lines indicate the median). SIRT: sirtuin; PGC-1α: peroxisome proliferator-activated receptor gamma coactivator 1 alpha; SREBP-1c: sterol regulatory element-binding protein, ChREBP: carbohydrate-responsive element-binding protein; MC: offspring of chow-fed dams; MHF: offspring of HFD-fed dams; MHFS: MHF offspring with SIRT1 overexpression. * *p <* 0.05, *** *p <* 0.001.

As can be seen from Figure 2A,B, WT offspring born to dams fed a HFD showed increased kidney weight (*p <* 0.001) and triglyceride levels (*p <* 0.01), which were reversed by SIRT1 overexpression in the MHFS mice (*p <* 0.01 and *p <* 0.05, respectively). No changes in the percentages of kidney weight per body weight were found. The mRNA and protein expression, as well as activity of SIRT1 in the offspring kidney in this study was significantly reduced by MHF (*p <* 0.05, Figure 2B). Consistently, the mRNA expression of PGC-1α was downregulated (*p <* 0.05) and that of ChREBP and SREBP-1c was upregulated (*p <* 0.05, Figure 2C). Increased expression of SIRT1 was confirmed in MHFS mice by both qRT-PCR and immunoblot (*p <* 0.001 and *p <* 0.05, respectively). SIRT1 activity were significantly increased in MHFS offspring (*p <* 0.05, Figure 2B). SIRT1 overexpression in MHFS offspring significantly increased PGC-1α expression and suppressed ChREBP (*p <* 0.01) but had no effect on SREBP-1c.

### 3.2. SIRT1 Overexpression Suppresses Renal Oxidative Stress and Inflammation Markers in MHF Offspring

**Figure 3:**
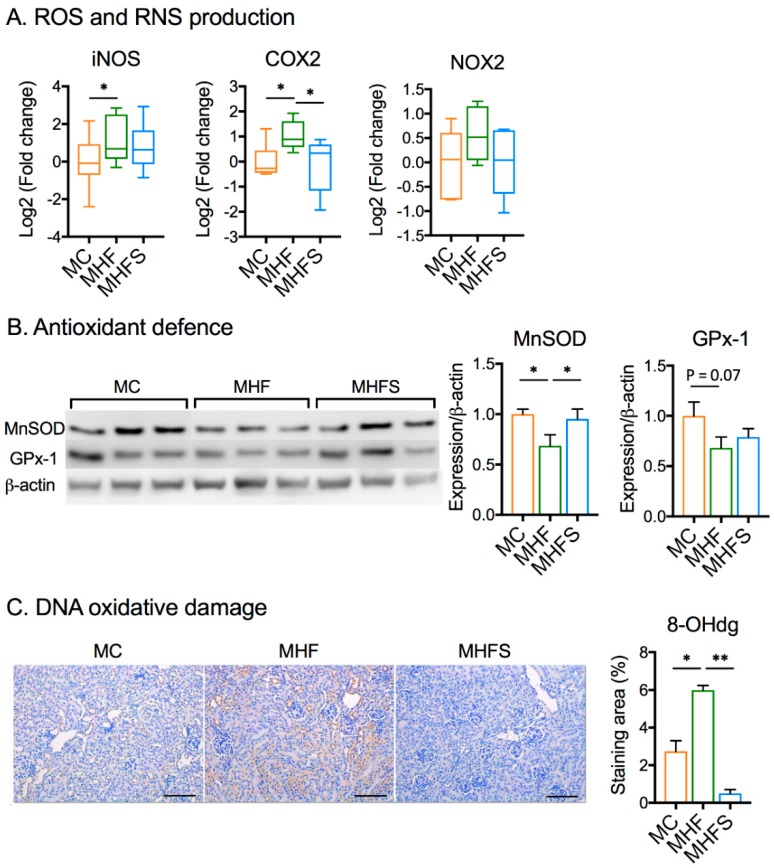
SIRT1 overexpression attenuates renal oxidative stress in offspring born to obese dams. (**A**) mRNA expression of oxidative stress markers (*n* = 6). (**B**) Protein expression of antioxidant enzymes. (**C**) Immunohistochemistry (IHC) staining and quantification of 8-hydroxy-2′-deoxyguanosine (8-OHdG) (*n* = 6). Data are presented as mean ± SEM or box plot (min to max, the central lines indicate the median). SIRT: sirtuin; iNOS inducible nitric oxide synthase; COX2: prostaglandin-endoperoxide synthase; NOX: NADPH oxidase; MnSOD: manganese superoxide dismutase; GPx: Glutathione peroxidase; MC: Offspring of chow-fed dams; MHF: offspring of HFD-fed dams; MHFS: MHF offspring with SIRT1 overexpression. * *p <* 0.05, ** *p <* 0.01.

WT offspring born to HFD-fed dams showed increased renal expression of inducible nitric oxide synthase (iNOS, *p <* 0.05) and prostaglandin-endoperoxide synthase (COX2, *p <* 0.05, Figure 3A), indicating increased production of reactive nitrogen and oxygen species (RNS and ROS, respectively). Concomitantly, the protein expression of antioxidant enzymes MnSOD but not GPx-1 was suppressed (*p <* 0.05, Figure 3B). 8-OHdG, which indicates oxidative damage of the DNA, was significantly increased due to MHF (*p <* 0.01, Figure 3C). SIRT1 overexpression in MHFS offspring normalised MnSOD protein expression (*p <* 0.05), and suppressed 8-OHdG accumulation (*p <* 0.05), suggesting reduced oxidative stress.

### 3.3. SIRT1 Overexpression Attenuates Renal Inflammation but not Albuminuria in MHF Offspring

**Figure 4:**
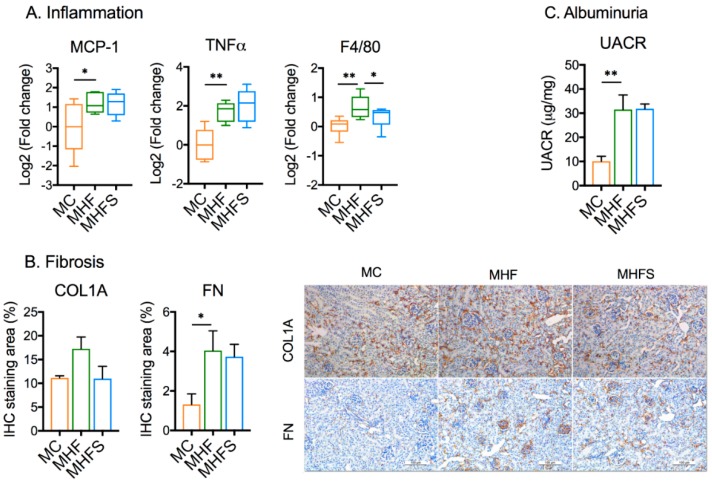
SIRT1 overexpression reduces markers of macrophage but not fibrosis and albuminuria in offspring born to obese dams. (**A**) mRNA expression of inflammation. (**B**) IHC staining quantification of fibrotic markers (*n* = 6). (**C**) Urinary albumin creatinine ratio UACR (*n* = 6). Data are presented as mean ± SEM or box plot (min to max, the central lines indicate the median). MCP: Macrophage chemotactic protein; TNFα: Tumour necrosis factor alpha; COL: collagen; FN: fibronectin. MC: offspring of chow-fed dams; MHF: offspring of HFD-fed dams; MHFS: MHF offspring with SIRT1 overexpression. * *p <* 0.05, ** *p <* 0.01.

The mRNA expression of inflammation markers including Macrophage chemotactic protein (MCP)-1 and Tumour necrosis factor alpha (TNF)α were upregulated in MHF offspring (*p <* 0.05, Figure 4A). The expression of F4/80, a macrophage marker, was also significantly elevated (*p <* 0.05, Figure 4A). SIRT1 overexpression significantly suppressed the expression of F4/80 (*p* < 0.05) but not MCP-1 and TNFα.

The protein expression of COL1A was not significantly changed despite a trend to increase at this early age (Figure 4B). In contrast, fibronectin FN deposition particularly in glomeruli was increased by MHF (*p <* 0.05), suggesting increased fibrogenesis. As a likely result, UACR was significantly elevated (*p <* 0.01, Figure 4C), reflecting albuminuria. Despite attenuation of oxidative and inflammatory markers, SIRT1 overexpression had no significant effects on the expression of both markers, as well as UACR.

### 3.4. SRT1720 Attenuates Renal Lipogenesis in Offspring Due to Maternal and Postnatal HFD Consumption

**Figure 5:**
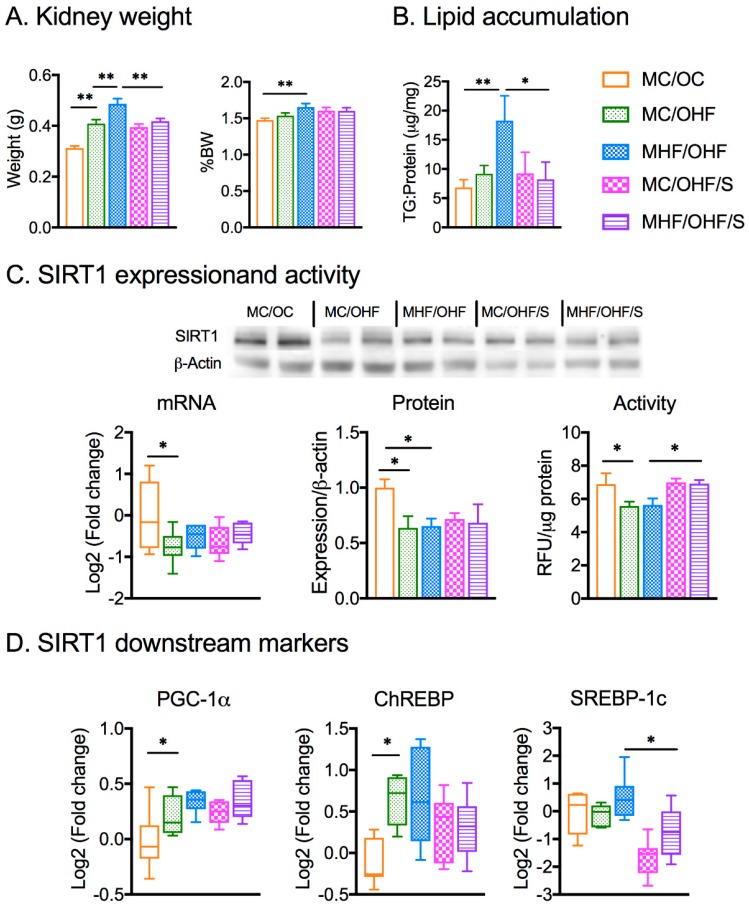
SRT1720 suppresses renal lipid accumulation in offspring due to maternal and postnatal HFD consumption. (**A**). Kidney weight (*n* = 9–17). (**B**) Kidney triglyceride level (*n* = 8). (**C**) SIRT1 expression and activity. (**D**) mRNA expression of SIRT1 downstream markers (*n* = 8). Data are expressed by mean ± SEM or box plot (min to max, the central lines indicate the median). SIRT: sirtuin; PGC-1α: peroxisome proliferator-activated receptor gamma coactivator 1 alpha; SREBP-1c: sterol regulatory element-binding protein, ChREBP: carbohydrate-responsive element-binding protein; MC: offspring of chow-fed dams; OC: chow-fed offspring; MHF: offspring of HFD-fed dams; OHF: HFD-fed offspring; S: SRT1720. * *p <* 0.05, ** *p <* 0.01.

At postnatal week 9, offspring fed a HFD for 6 weeks (OHF) had increased kidney net weight (*p <* 0.01) in comparison to chow-fed offspring (Figure 5A). Maternal HFD consumption exacerbated this effect (*p <* 0.01). Kidney weight/body weight ratio and kidney triglyceride levels were only increased in offspring exposed to both maternal and postnatal HFD (*p <* 0.05, Figure 5B), suggesting the significant contribution of maternal HFD consumption to offspring’s kidney lipid metabolism. SIRT1′s mRNA expression, protein expression, and activity were significantly decreased due to OHF (*p <* 0.05, Figure 5C). There was no further reduction in MHF/OHF offspring. In contrast to our expectation, PGC-1 α expression was increased by OHF (*p <* 0.05, Figure 5D). ChREBP mRNA expression was significantly increased in MC/OHF offspring (*p <* 0.05). SREBP-1c levels were unchanged. There was no additive effects from MHF on the regulation of these markers. 

SRT1720 administration significantly reduced kidney net weight (*p <* 0.01, Figure 5A) and renal level of triglycerides (*p <* 0.05, Figure 5B) in MHF/OHF/S offspring. As expected, the agonist did not increase mRNA or protein expression of SIRT1 but normalised its activity in these offspring (*p <* 0.05 Figure 5C). Such increase was associated with reduced mRNA expression of SREBP-1c (*p <* 0.05, Figure 5D). However, no significant changes were found in the expression of the other two downstream markers.

### 3.5. SRT1720 Suppresses Renal Oxidative Stress and Inflammation Markers in Offspring Due to Maternal and Postnatal HFD Consumption 

**Figure 6:**
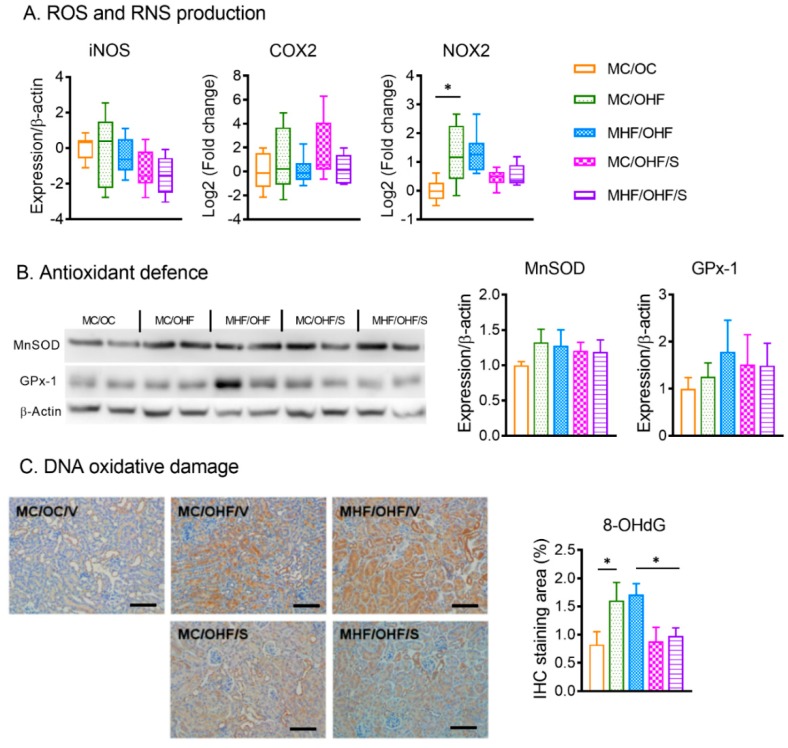
SRT1720 attenuates renal oxidative stress in offspring due to maternal and postnatal HFD consumption. (**A**) mRNA expression of oxidative stress markers (*n* = 8). (**B**) Protein expression of antioxidant enzymes (*n* = 8). (**C**) IHC staining and quantification of 8-hydroxy-2′-deoxyguanosine (8-OHdG) (*n* = 8). Data are presented as mean ± SEM or box plot (min to max, the central lines indicate the median). iNOS inducible nitric oxide synthase; COX2: prostaglandin-endoperoxide synthase; NOX: NADPH oxidase; MnSOD: manganese superoxide dismutase; GPx: Glutathione peroxidase; MC: offspring of chow-fed dams; OC: chow-fed offspring; MHF: offspring of HFD-fed dams; OHF: HFD-fed offspring; V or VEH: vehicle control, S or SRT: SRT1720. * *p <* 0.05.

In contrast to P20, no changes in mRNA expression of iNOS and COX2 in the offspring kidney were found in the offspring born to obese dams and/or fed a postnatal HFD in comparison to the control MC/OC (Figure 6A). However, the expression of NADPH oxidase (NOX)2 was significantly increased (*p <* 0.01). On the other hand, MnSOD and GPx-1 protein expression was not significantly changed, demonstrating a trend to adaptation of antioxidant defence in the offspring in adolescent following postnatal HFD exposure (Figure 6B). 8-OHdG accumulation was significantly higher in the offspring from HFD-fed dams compared to the control group (*p <* 0.01, Figure 6C), reflecting increased DNA oxidative damage despite normal antioxidant levels. SRT1720 treatment significantly suppressed renal expression of NOX2 in MHF/OHF/S offspring (*p <* 0.05). In line with NOX2 expression, the levels of 8-OHdG were significantly suppressed by SRT1720 treatment (*p <* 0.05, Figure 6B), suggesting ameliorated oxidative stress.

### 3.6. SRT1720 Attenuates Renal Fibrogenesis but not Albuminuria in Offspring Due to Maternal and Postnatal HFD Consumption

**Figure 7:**
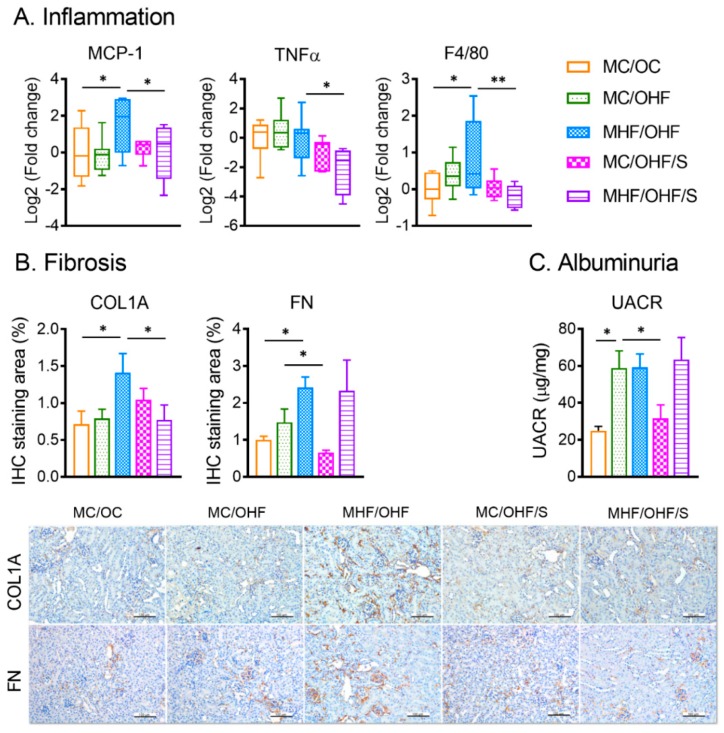
SRT1720 attenuates renal inflammation and fibrosis but not the urinary albumin creatinine ratio in offspring due to maternal and postnatal HFD consumption. (**A**) mRNA expression of inflammatory markers. (**B**) Immunohistochemistry quantification of fibrotic markers (*n* = 8). (**C**) Urinary albumin creatinine ratio UACR (*n* = 8). Data are presented as mean ± SEM. MCP: Macrophage chemotactic protein; TNFα: Tumour necrosis factor alpha; COL: collagen; FN: fibronectin. MC: offspring of chow-fed dams; OC: chow-fed offspring; MHF: offspring of HFD-fed dams; OHF: HFD-fed offspring; S: SRT1720. * *p <* 0.05, ** *p <* 0.01.

Maternal HFD consumption significantly increased the expression of inflammation marker MCP-1 (vs MC/OHF, *p <* 0.05) and macrophage marker F4/80 (vs MC/OC, *p <* 0.05) in the offspring (Figure 7A). SRT1720 significantly suppressed MCP-1 (*p <* 0.05), TNFα (*p <* 0.01), and F4/80 (*p <* 0.05) mRNA levels. Consistent with the results at weaning, maternal HFD consumption significantly exacerbated renal extracellular matrix (ECM) deposition of COL1A (*p <* 0.05) and FN (*p <* 0.05) in offspring in adolescence (Figure 7B). Such an effect was not evident in animals exposed to postnatal HFD only. SRT1720 administration suppressed the expression of COL1A (*p <* 0.05) but not FN (*p <* 0.05) in MHF/OHF offspring. In line with the changes of fibrotic markers, UACR was significantly elevated by postnatal HFD with and without maternal HFD pre-exposure (*p <* 0.01, Figure 7C). The increase was modulated by SRT1720 treatment only in MC/OHF/S offspring (*p <* 0.05).

## 4. Discussion

Maternal obesity is a risk factor of metabolic disorders such as diabetes and hyperlipidaemia in the offspring, which in turn can contribute to the early development and progression of CKD. In the present study, we confirm that maternal high-fat consumption-induced obesity can lead to increased lipid accumulation, oxidative stress, inflammation, and fibrosis in the offspring at weaning and adolescence. Importantly, these pathological changes are associated with reduced SIRT1 expression and activity. The upregulation of SIRT1 signalling by means of genetic modification or administration of SIRT1 agonist SRT1720 is found to attenuate renal lipid deposition, oxidative stress, and inflammation but not fibrosis and albuminuria due to maternal obesity. 

It is noteworthy that we have also examined metabolic disorders in the same model and showed that offspring born to obese dams had increased body weight, hyperlipidaemia and hyperglycaemia at weaning [13] and adolescence [22], which were significantly reversed by SIRT1 overexpression/activation. As these metabolic disorders are important risk factors of CKD, the improvements in systemic glucose and lipid metabolism by SIRT1 may also contribute to the attenuated kidney disorders due to maternal HFD in the offspring. A future study using kidney-specific SIRT1 overexpression mice is required to clarify its direct and indirect effects.

In kidney, SIRT1 overexpression in MHFS offspring led to the recovered expression of PGC-1α, as well as the suppression of ChREBP. On the other hand, SRT1720 administration suppressed SREBP1 expression. PGC-1α is involved in mitochondrial biosynthesis and fatty acid oxidation [23,24]. Defective expression of fatty acid oxidation genes including PGC-1α in renal tubular epithelial cells has been implicated in the development of kidney fibrosis [25,26]. ChREBP and SREBP1 have been shown to be up-regulated in the kidney of diabetic mice [27,28,29]. The ablation of either protein was able to prevent lipotoxicity, oxidative stress, inflammation, fibrosis, and albuminuria. The results provide corroborative evidence as to the significance of SIRT1 signalling in the regulation of renal lipotoxicity and nephropathy due to maternal obesity.

Oxidative stress is induced by the imbalance between the production of reactive oxygen/nitrogen species (ROS/RNS) and antioxidant capacity. Elevated oxidative stress has been suggested to be the major instigator of diabetic nephropathy [30], leading to glomerular inflammation and tubulointerstitial fibrosis. In this study, in concert with increased lipid accumulation, MHF offspring have elevated renal oxidative stress, as reflected by the increased levels of iNOS, COX2, and 8-OHdG, as well as reduction of MnSOD at weaning. In adolescence, NOX2 and 8-OHdG were elevated. The increases in iNOS and 8-OHdG are consistent with our previous studies [8,31]. Particularly, iNOS has been characterised as one of the earliest effects of diabetes on kidney [32]. iNOS is a major source of nitric oxide (NO), while NO tends to be deficient in the advanced stages of CKD [33], which may explain the unchanged expression of iNOS at week 9. MnSOD levels were recovered at week 9, likely due to a compensatory effect to prolonged oxidative stress. The result is in line with a previous study by Ruggerio et al. [34], in which they fed C57BL/6 mice a HFD for 12 or 16 weeks and found an adaptation of mitochondrial biogenesis, antioxidant and respiratory machinery in the kidney despite the evident of oxidative stress. Indeed, PGC-1α, the pivotal regulator of mitochondrial biogenesis, was also increased in the offspring following postnatal HFD exposure in our study. No additive effects were induced by maternal HFD consumption with regard to oxidative stress markers, suggesting the parts of the maternal effects have been overwhelmed by postnatal HFD. Such phenomenon has been demonstrated in our previous studies [35]. 

SIRT1 overexpression suppressed COX2 levels at weaning, which is in line with the study by Jung et al. showing a downregulation of COX2 in aged rat kidney following short-term caloric restriction [36]. In addition, SIRT1 overexpression/activation also normalised MnSOD expression and 8-OHdG accumulation at weaning, and suppressed NOX2 and 8-OHdG levels at week 9, thus confirming the protective effects of SIRT1 therapy against maternal obesity-induced kidney oxidative damage at different developmental stages. SIRT1 overexpression and activation had no effects on iNOS expression, which may suggest a less potent role of SIRT1 in modulation of nitrosative stress. The results also imply differences between SIRT1 overexpression and SRT1720 in their mechanisms to regulate oxidative stress.

Apart from oxidative stress, the study also showed significant increases in inflammatory markers including MCP-1 and F4/80. The elevated levels of renal fibrosis markers including COL1A and FN and albuminuria in offspring of HFD-fed dams at both weaning and week 9 are consistent with our previous reports, clearly confirming that maternal obesity contributes to the risk of CKD in offspring [8,31]. SIRT1 overexpression significantly suppressed F4/80, and SRT1720 treatment reduced MCP-1, TNFα, and F4/80 in the offspring exposed to maternal HFD, suggesting the anti-inflammatory effects of SIRT1 against maternal obesity-induced kidney inflammation. Although SIRT1 activation suppresses COL1A deposition in MHF/OHF/S offspring kidney in adolescence, FN levels were not affected. Similarly, although albuminuria by SRT1720 was reduced in the offspring on postnatal HFD, which is consistent with a previous study in diabetic animals [19], it persisted in those pre-exposed to maternal high-fat diet, suggesting that offspring affected by maternal obesity are likely to resist SIRT1 therapy regarding albuminuria. A combination with gestational weight control and other therapies may be necessary to increase the effectiveness. Besides, natural SIRT1 activators such as resveratrol, or NAD+precursors can be tested on humans to further investigate the clinical relevance of the therapy [37].

Collectively, the study demonstrates that SIRT1 plays essential roles in kidney programming, upregulation of which can partially suppresses renal lipid accumulation, oxidative stress, inflammation, and fibrogenesis in the offspring born to obese mothers. However, further studies are required to address persistent albuminuria.

## Abbreviations

8-OHdg	8-hydroxy-2′-deoxyguanosine
ACEC	Animal Care and Ethics Committee
ChREBP	Carbohydrate response-element-binding protein
COL	Collagen
COX	Prostaglandin-endoperoxide synthase
ECM	Extracellular matrix
F4/80	Marker of macrophage
FN	Fibronectin
iNOS	Inducible nitric oxide synthase
HFD	High-fat diet
MCP-1	Macrophage chemotactic protein 1
MHF	Maternal high-fat diet consumption
NOX	NADPH oxidase
OHF	High-fat feeding in offspring
PGC-1α	Peroxisome proliferator-activated receptor gamma coactivator 1-α
PPAR	Peroxisome proliferator-activated receptors
ROS	Reactive oxygen species
SIRT	Silent information regulator
S	SRT1720
SREBP	Acetylated sterol regulatory element binding proteins
TNFα	Tumour necrosis factor α
UACR	Urine albumin/creatinine ratio
V	Vehicle control
